# Double Trouble: An Unusual Presentation of Bilateral Penile Fracture

**DOI:** 10.7759/cureus.63440

**Published:** 2024-06-29

**Authors:** Kishor M, Deepak Ghuliani, Sushanto Neogi, Shiva Kiran Addu, Abhinav Singh

**Affiliations:** 1 General Surgery, Maulana Azad Medical College, New Delhi, IND

**Keywords:** penile deformity, sexual intercourse, double penile fracture, aast grading of penile fracture, penile fracture

## Abstract

A penile fracture results from the rupture of the tunica albuginea due to blunt trauma to an erect penis and is a rare urological emergency. Double penile fractures involving both corpora cavernosa injuries are extremely uncommon. We report the case of a 38-year-old male who experienced acute penile pain, swelling, and a "cracking" sound during sexual intercourse. Examination and ultrasound confirmed bilateral tunica albuginea ruptures and hematoma. The surgical repair involved hematoma evacuation and suturing of the tears. The patient recovered without complications. This case highlights the necessity for prompt recognition and surgical intervention in double penile fractures to prevent long-term complications and ensure optimal recovery.

## Introduction

Penile fracture is an uncommon but serious urological condition resulting from the rupture of the tunica albuginea due to blunt trauma to an erect penis [1]. The tunica albuginea is a tough fibrous layer that surrounds the corpora cavernosa, and its rupture typically occurs during vigorous sexual intercourse or masturbation. In the United States, the estimated incidence is approximately 1 in 175,000 hospital admissions annually [2].

Double penile fractures, involving simultaneous ruptures of both corpora cavernosa, are exceedingly rare. There is limited literature on the exact incidence, but it is considered to be less than 2% of all penile fracture cases [3]. This report presents a unique case of a 38-year-old male with a double penile fracture following sexual intercourse.

## Case presentation

**Figure 1:**
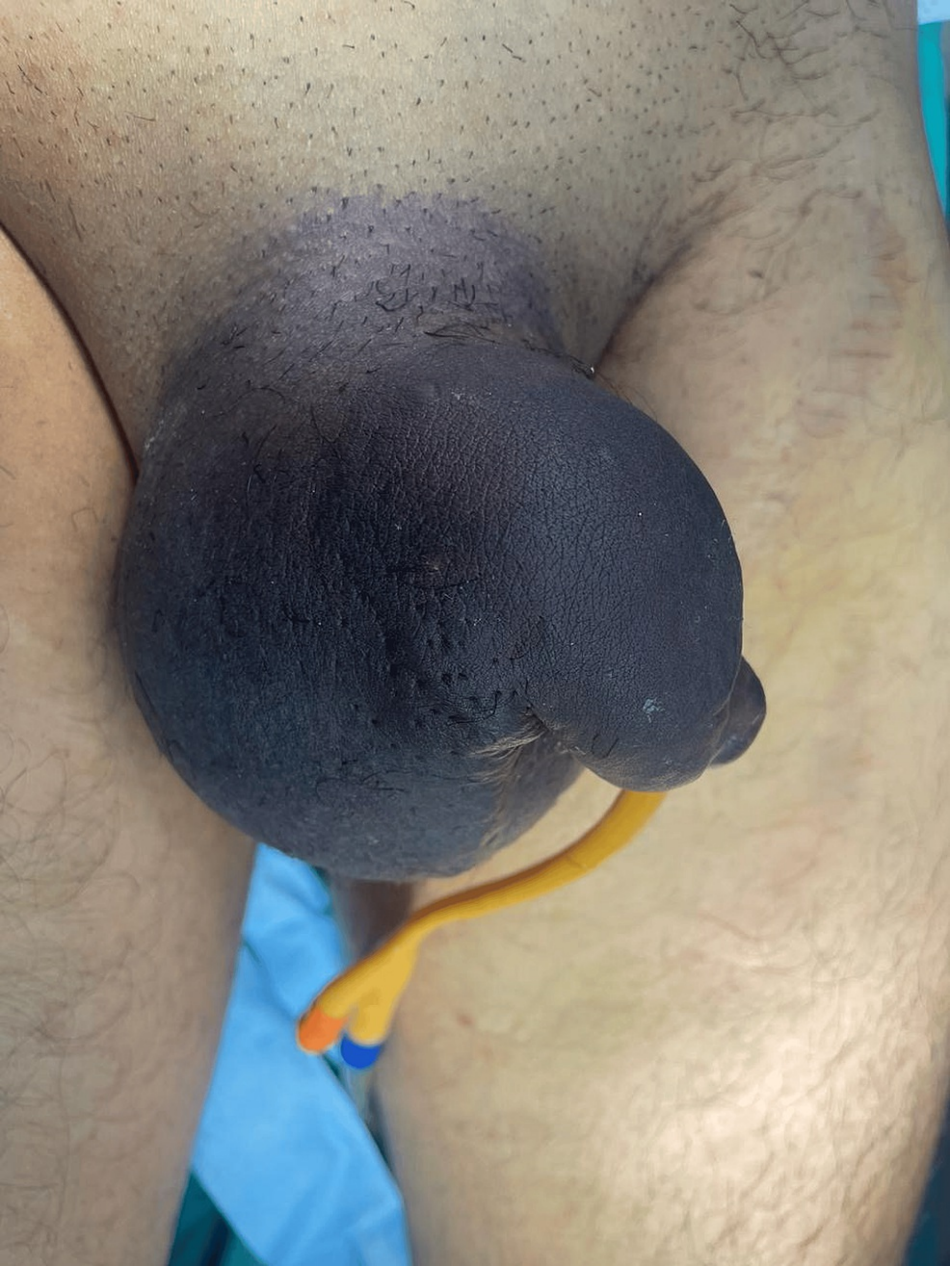
Penile deformity with penile and scrotal hematoma

**Figure 2:**
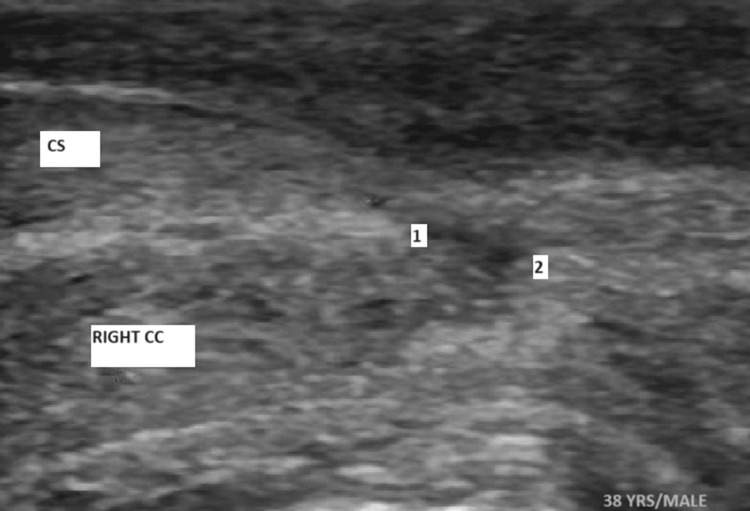
Ultrasonographic image showing a defect in the tunica albuginea (between 1 and 2) of right CC Points 1 and 2 represent the defect in the tunica albuginea. CC: Corpus cavernosum, CS: Corpus spongiosum

A 38-year-old male presented to the emergency department 14 hours after experiencing acute penile pain and swelling during sexual intercourse. He reported hearing a sudden "cracking" sound, followed by immediate pain, penile deformity, and scrotal edema. Physical examination revealed a swollen, deviated penis with notable hematoma and scrotal edema with no blood at the tip of the urethral meatus, features suggestive of penile fracture (Figure 1). Urgent penile ultrasound also confirmed disruption of the tunica albuginea near the root of the penis, with a substantial hematoma present, no urethral injury was detected (Figure 2).

**Figure 3:**
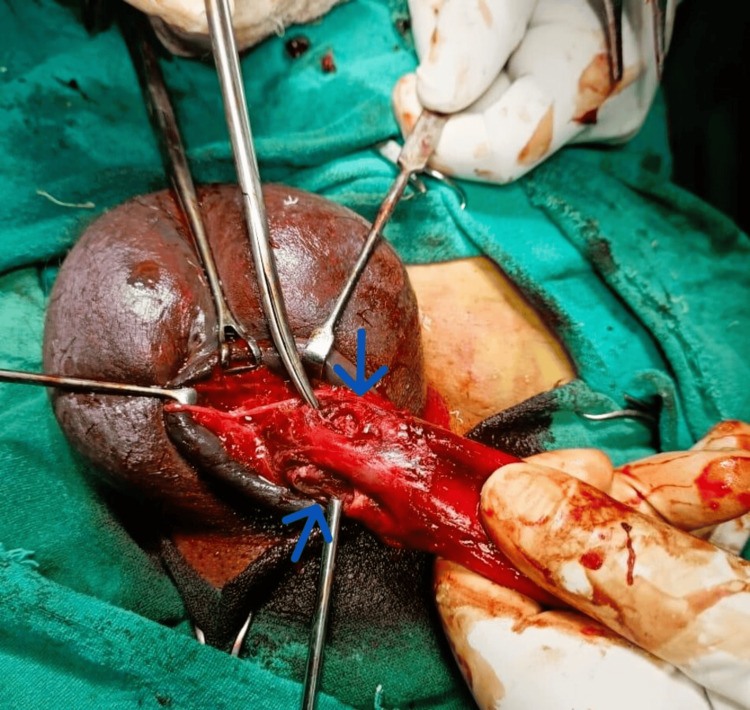
Bilateral cavernosal tear near penile root (arrows)

**Figure 4:**
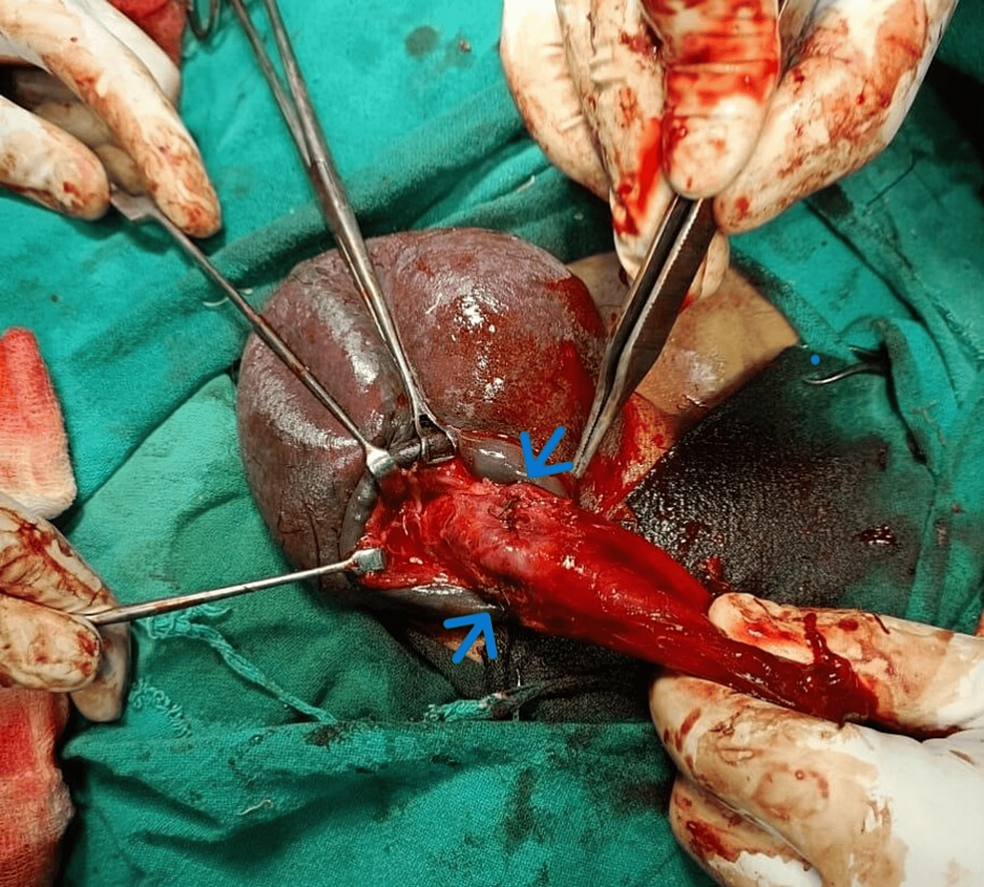
Post repair of bilateral tunica albuginea near root of penis using vicryl 3–0 sutures (arrows)

The patient was taken to the operating room for urgent surgical exploration. A circumferential subcoronal degloving incision was performed, exposing the penile shaft. Intraoperatively, there were bilateral corpus cavernosal tears near the penile root with hematoma with American Association for the Surgery of Trauma (AAST) grade 2 for penile fracture (Figure 3). The hematoma was meticulously evacuated, and the bilateral tears in the tunica albuginea were repaired using vicryl 3-0 absorbable sutures with a simple interrupted technique (Figure 4).

**Figure 5:**
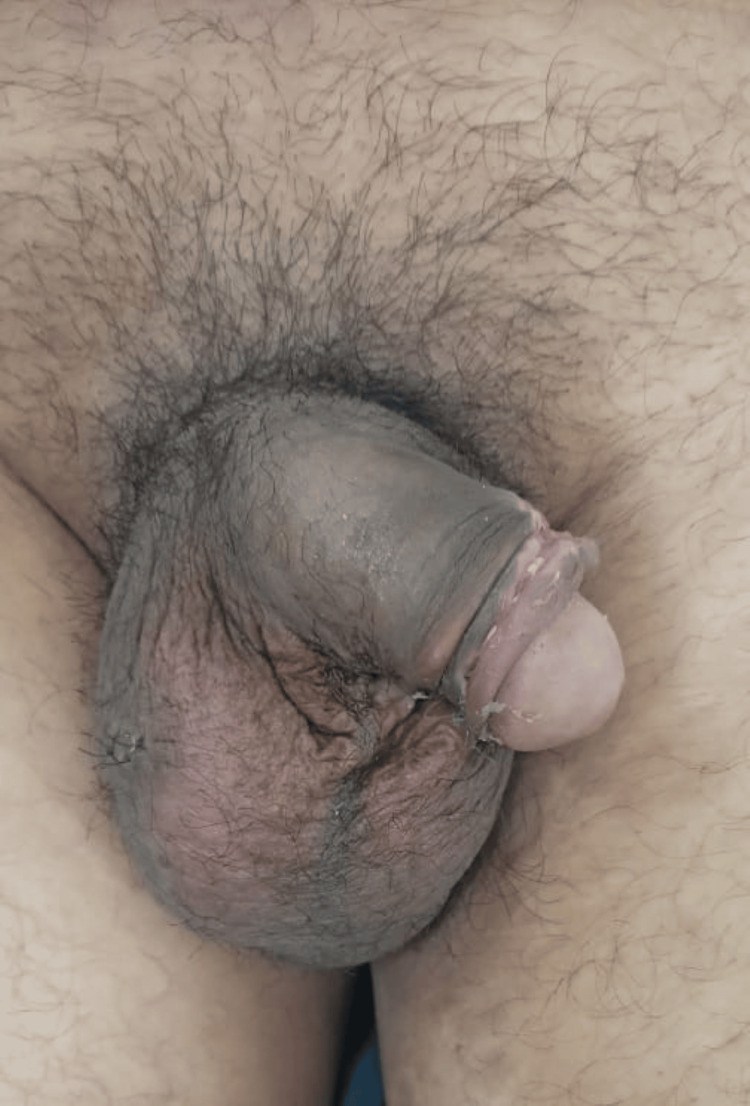
Third-week follow-up showing significant reduction in edema of scrotum and penis with the suture line appearing healthy

Postoperatively, the patient was discharged with a prescription for amoxicillin-clavulanate 625 mg to be taken thrice daily for seven days, along with a Foley catheter in situ. He was instructed to abstain from sexual activity and masturbation for at least four weeks and was educated on performing daily home dressing. The Foley catheter was subsequently removed after one week. At the third-week follow-up, the surgical site appeared healthy and free of discharge, and the patient reported no penile curvature during erection. He expressed satisfaction with the overall outcome (Figure 5).

## Discussion

Penile fracture typically presents with immediate penile pain, swelling, and hematoma following trauma to an erect penis [4]. The incidence of penile fracture varies globally but is generally considered rare. Higher incidences have been reported in regions where cultural practices involve vigorous sexual activity or aggressive penile manipulation [5]. Bilateral corpus cavernosal tears, although rare, present similarly but may involve more extensive hematoma and deformity. The exact incidence of double penile fractures is not well documented, but it is believed to be less than 2% of all penile fracture cases [3].

The AAST has developed a grading system for penile fracture to help standardize the severity of the injury and guide treatment [6]. Table 1 explains the AAST grading of penile injury.

**Table 1:** The AAST grading for penile injury AAST: American Association for the Surgery of Trauma

Grade	Description
1	Contusion or hematoma without urethral injury
2	Laceration involving the tunica albuginea without urethral injury
3	Laceration involving the tunica albuginea with urethral injury
4	Laceration involving the tunica albuginea with injury to the corpus spongiosum
5	Complete disruption of the penis including urethral injury

The algorithm for diagnosing penile fracture begins with clinical evaluation, which includes assessing the patient's history of trauma events and conducting a physical examination to check for penile swelling, ecchymosis, and deformity. An initial diagnosis is based on clinical suspicion derived from the history and physical exam [1]. If the diagnosis is uncertain or a urethral injury is suspected, imaging studies are recommended. Ultrasound, a non-invasive modality, can confirm the presence of tunica albuginea tears and detect hematoma [7]. An MRI offers high-resolution images of soft tissue structures, though it is less commonly used due to higher cost and limited availability in emergency settings [8]. By following this algorithm, healthcare providers can effectively diagnose and manage penile fractures.

Prompt surgical intervention is the mainstay of treatment for penile fractures to prevent long-term complications such as erectile dysfunction and penile curvature. The goal of surgery is to evacuate the hematoma, repair the tunica albuginea tear(s), and address any associated injuries, such as urethral damage [9]. Absorbable sutures are typically used to repair the tunica albuginea to minimize foreign body reactions and enhance healing [10].

This case highlights the importance of considering bilateral penile fracture in patients presenting with severe penile trauma and deformity. Surgical repair involves evacuation of the hematoma and meticulous suturing of the tunica albuginea to restore penile integrity and function.

## Conclusions

A double penile fracture is a rare but serious urological emergency requiring prompt recognition and surgical management. This case demonstrates successful treatment through timely intervention, resulting in favorable functional outcomes. Clinicians should maintain a high index of suspicion for bilateral fractures in cases of significant penile trauma to ensure optimal patient recovery.
